# Distribution of Ugandan Passiflora Virus (*Potyvirus passiflorafricanse*) in Major Passion Fruit Growing Areas in Rwanda

**DOI:** 10.3390/v18030397

**Published:** 2026-03-23

**Authors:** Esperance Munganyinka, Bancy W. Waweru, Marie Claire Kanyange, Josiane Umubyeyi, Ghislain Niyonteze, Lydie Kankundiye, Melanie Mukashimwe

**Affiliations:** 1 National Council for Science and Technology (NCST), Kigali P.O. Box 2285, Rwanda; 2 Rwanda Agriculture and Animal Resources Development Board (RAB), Kigali P.O. Box 5016, Rwanda; bancy.waweru@rab.gov.rw (B.W.W.); marieclaire.kanyange@rab.gov.rw (M.C.K.); mujosiene08@gmail.com (J.U.); ghislain.niyonteze@rab.gov.rw (G.N.); lydie.kankundiye@rab.gov.rw (L.K.); melanie.mukashimwe@rab.gov.rw (M.M.)

**Keywords:** *Passiflora edulis* Sims, detection, virus, passion fruit woodiness disease, East Africa

## Abstract

Passion fruit (*Passiflora edulis* Sims) is an important economic fruit crop in Rwanda grown for both domestic consumption and export markets. However, viral diseases pose a significant threat to passion fruit production. Among these, passion fruit woodiness disease (PWD) is the most destructive, causing yield losses of up to 100%. A survey was carried out to assess the distribution of Ugandan passiflora virus (UPV; *Potyvirus passiflorafricanse*) in major passion fruit growing areas. UPV is one of the major viruses known to cause PWD. The incidence of viral symptoms observed in the field did not differ significantly among districts, ranging from 81% in Rusizi to 100% in Rwamagana. However, mean symptom severity scores varied significantly between districts, with the highest severity recorded in Kayonza (3.1) and the lowest in Rulindo (1.9). Serological analysis detected potyviruses in 44% of the total samples (*n* = 216), including 43% of symptomatic (*n* = 144) and 47% of asymptomatic (*n* = 72) leaf samples collected from passion fruit fields. Further analysis using Reverse-Transcription Polymerase Chain Reaction (RT-PCR) detected UPV in 56% of symptomatic (*n* = 126) and 53% of asymptomatic (*n* = 60) samples, corresponding to 55% of the total samples tested (*n* = 186). The virus was present in all surveyed districts, with UPV infection prevalence of 89% in Rusizi, 75% in Rwamagana, 74% in Karongi, 59% in Nyamagabe, 44% in Nyaruguru, 38% in Kayonza, and 30% in both Gakenke and Rulindo. Fifteen partial coat-protein gene sequences for the Rwandan isolates were obtained. The newly described Rwandan isolates shared 97–99% nucleotide (nt) identity with one another, 89–94% with previously reported Rwandan isolates, 81–97% with Ugandan isolates, and 80–82% with Kenyan UPV isolates, suggesting that the Rwandan virus population is relatively homogenous. Genetic distances among the 15 new UPV isolates and previously reported Rwandan, Ugandan, and Kenyan isolates were very short (0.01–0.03), indicating high sequence similarity. All Rwandan isolates clustered into a single major clade, together with some Ugandan and Kenyan isolates. This close genetic relationship suggests a common ancestry and the regional spread of a single dominant UPV lineage. These findings highlight the need to reinforce seed and planting-material certification systems, as well as the need to enhance farmer capacity through targeted training on viral disease identification and management practices. This is vital to limiting the spread of viral diseases that threaten income security among smallholder passion fruit farmers.

## 1. Introduction

Passion fruit (*Passiflora edulis* Sims), which originated in the tropical and subtropical regions of South America, is now widely cultivated globally. It belongs to the *Passifloraceae* family, with a wide genetic base of approximately 525 species [1]. Among more than 60 species of the passion fruit genus (*Passiflora*) producing edible fruits, the most important include purple passion fruit (*Passiflora edulis f. edulis* Sims), yellow passion fruit (*Passiflora edulis f. flavicarpa* Deg.), sweet granadilla (*Passiflora ligularis:* Juss), and granadilla (*Passiflora quadrangularis* L.) [2]. In Africa, the crop holds significant economic and nutritional importance in many countries, providing income for smallholder farmers and contributing to local diets with essential minerals, vitamins, phytoconstituents, phenolic compounds, and fiber [3,4].

In Rwanda, passion fruit is currently one of the priority crops for export diversification, with production expected to increase from 2523MT in 2023 to 5783MT in 2028 [5]. About 46% of the crop is grown in the western, 43% in the northern, 10% in the southern and 1% in the eastern part of the Rwanda [6]. It contributes significantly to household income, nutrition, and export earnings. Passion fruit export revenues increased 1.7 fold from $245,712 in 2022/2023 to $426, 573 US dollar in 2023/2024 [7]. In spite of its economic importance, viruses are a major cause of diseases in passion fruit production and pose a significant threat to fruit yields and quality [4]. In addition, diseases have been documented to reduce the life span of passion fruit from 7 years to a mean of 1 to 2 years [8,9].

More than 25 different viruses belonging to the *Potyvirus*, *Cucumovirus*, *Begomovirus*, *Tymovirus*, *Cilevirus*, *Carlavirus*, *Tobamovirus*, and *Nepovirus* genera have been identified and characterized in passion fruit plants [10,11,12]. Passion fruit woodiness disease is one of the major constraints on its production in Rwanda and other passion fruit growing countries, where it causes severe yield and quality losses [10,13,14]. The disease is predominantly associated with infection by potyviruses [8]. It is widely spread, and symptoms associated with the disease include leaf mosaic and hard fruits [8]. Globally, pathogens documented to cause passion fruit woodiness disease include the East Asian passiflora virus (EAPV), cowpea aphid-borne mosaic virus (CABMV), passion fruit woodiness virus (PWV) and Ugandan passiflora virus (UPV). In the East Africa region, pathogens associated with PWD include CABMV, as reported in Kenya and Rwanda, and UPV, as reported in Uganda, Kenya and Rwanda [10,12,15].

Ugandan passiflora virus is a potyvirus that was first characterized in Uganda as a distinct species based on coat-protein sequencing, symptomatology, and host reactions [8]. A subsequent survey confirmed its presence in Rwanda, where a high proportion of symptomatic passion fruit plants tested positive for UPV [14]. Although UPV was previously reported in Rwanda in 2018, the study was not extensive and considered few passion fruit growing districts. Virus populations and disease dynamics are known to change over time due to shifts in host distribution, vector pressure, and agricultural practices [16]. The current study aimed at determining the distribution of UPV in major passion fruit growing areas across different agro-ecological zones in Rwanda. Understanding the distribution of UPV is therefore crucial for the design of effective disease management strategies and to safeguard passion fruit production.

## 2. Materials and Methods

### 2.1. Study Areas

**Figure 1:**
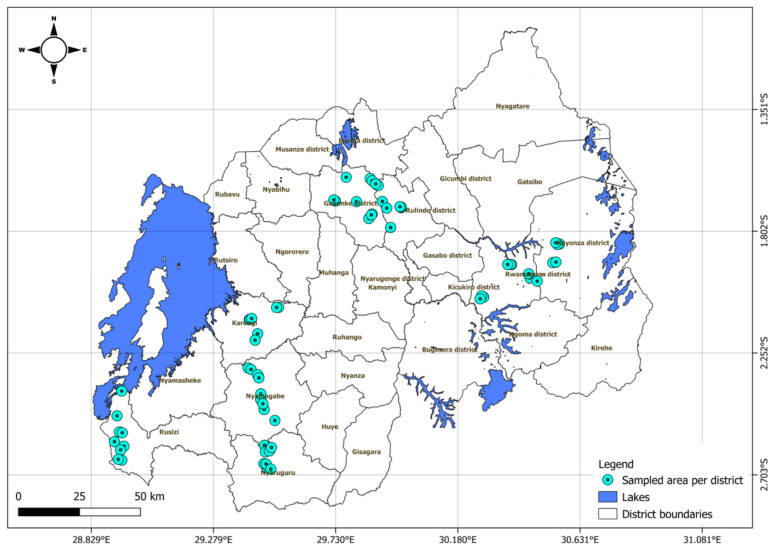
Map of Rwanda showing passion fruit growing areas and districts that were sampled for woodiness disease.

Passion fruit leaf samples were collected from major growing areas covering low- and high-altitude areas from July to September 2021. Based on areas where passion fruit is mainly cultivated, eight districts were purposively selected in four provinces: Nyamagabe and Nyaruguru in Southern Province, Gakenke and Rulindo in Northern Province, Rusizi and Karongi in Western Province, and Rwamagana and Kayonza districts in Eastern Province (Figure 1). Rwamagana and Kayonza represent low-altitude zones, while areas sampled in Nyamagabe, Nyaruguru, Gakenke, Rulindo, Karongi, and Rusizi fall within high-altitude agro-ecological zones (AEZs).

### 2.2. Field Assessment of Incidence, Severity and Prevalence of Viral Diseases

A total of 72 passion fruit fields across the districts were visited, among which nine fields were randomly selected per district. In each field, in an area of 20 m by 20 m, twenty plants were randomly examined along 2 diagonals (x-shaped transect stretching between opposite corners) for virus incidence and severity. Disease incidence was determined by considering the ratio of the number of plants with symptoms to the total number of plants examined, expressed as a percentage [16]. Disease severity was determined using a scale of 1 to 5, where 1 = no symptoms; 2 = mild mosaic and no leaf deformation; 3 = moderate mosaic, blisters and leaf deformation; 4 = severe mosaic, blisters, leaf and fruit deformation, and stunting; and 5  =  very severe mosaic, blisters, leaf and fruit deformation, severe stunting, and plant death [8]. Disease prevalence was estimated as the proportion of fields with disease symptoms expressed as a percentage of the total number of fields visited [8].

### 2.3. Collection of Symptomatic and Asymptomatic Passion Fruit Leaf Samples

Systematic sampling was conducted in all selected fields. The fields were at least 3–5 km apart, contained more than 50 plants, and had crops older than six months. From each field, two samples were randomly collected from symptomatic plants showing virus-like symptoms such as mosaic, blisters, distorted leaves, and stunting and carrying small, hardened, cracked fruits. Each sample was collected from a separate plant, targeting 3–5 expanding leaves from different growing points of the vines. In addition, a third sample from a symptom-less plant was collected from each field as a negative control. In total, 216 samples were collected and placed in envelopes containing silica gel. The samples were transported to the phytopathology laboratory of the Rwanda Agriculture and Animal Resources Development Board (RAB) at Rubona Station, Huye District, where they were stored at room temperature for 4–5 days until properly dried. This was followed by grinding of the samples into a fine powder in liquid nitrogen, which was stored −80 °C until analyzed.

### 2.4. Serological Assays

All collected samples (216) were tested for the presence of generic potyviruses using a commercial Plate-Trapped Antigen Enzyme-Linked Immunosorbent Assay (PTA-ELISA) kit (Catalogue POTY-XRA0500, Sediag, Bourgogne-Franche-Comté, France). In addition, the samples were tested for CABMV (Catalogue AS-0417) and CMV (Catalogue AS-0929) using Double Antibody Sandwich (DAS)-ELISA kits from Leibniz Institute DSMZ, Germany. All assays were performed according to manufacturer protocols. Absorbance values were read at 405 nm using a microplate reader (BioTek ELX800, Winooski, VT, USA). Samples with absorbance values greater than two times that of the average of negative controls were rated as positive. The buffers and negative and positive controls were provided with the kits.

### 2.5. Extraction of Ribonucleic Acid (RNA) and Reverse-Transcription Polymerase Chain Reaction (RT-PCR)

To confirm the presence of viruses from ELISA positives and to identify UPV, a potyvirus for which a commercial antisera kit is not available, RT-PCR tests were done. One hundred eighty-six (186) samples, including all ELISA positives (96) and some negatives (90), were tested. Approximately 100 mg of frozen, powdered passion fruit leaf tissue from each sample was used to extract total RNA using a Cetyltrimethylammonium bromide (CTAB) procedure [17]. The concentration, quality and integrity of RNA were checked using Nanodrop (NanoDrop™ 2000, Thermo Fisher Scientific, Waltham, MA, USA) and gel visualization (BioDoc-It^®^ Imaging System, Ultra-Violet Products Ltd., Upland, CA, USA). In this study, previously designed virus-specific primers targeting the coat-protein region of Uganda passiflora virus were used to amplify a 772 bp fragment [14]. With this aim, a one-step Access RT-PCR kit (Catalogue A1250, Promega Corporation, Madison, WI, USA) was used following the manufacturer’s protocol. The RT-PCR amplification mixture comprised 10 μL of AMV/Tfl 5× Reaction buffer,1 μL of dNTP mix (10 mM each dNTP), 1 μL of 10 μm forward primer, 1 μL of 10 μm reverse primer, 2 μL of 25 mM MgSO_4_, 1 μL of AMV reverse transcriptase (5 u/μL), 1 μL of Tfl DNA polymerase (5 u/μL), and 1 μL of total RNA, with the reaction mix brought to a final volume of 50 μL with nuclease-free water. Reverse transcription was performed at 45 °C for 45 min, followed by AMV RT inactivation and RNA/cDNA/primer denaturation at 94 °C for 2 min, denaturation for 40 cycles of 94 °C for 30 s, annealing at 48 °C for 1 min, extension at 68 °C for 2 min, and a final extension at 68 °C for 7 min.

DNA fragments were separated by electrophoresis in a 1.2% (*w*/*v*) agarose gel containing 0.4 µg/mL of ethidium bromide at 135 V for 30 min in 1 × Tris–Acetate-EDTA (TAE) buffer. Gels were visualized under UV light using Gel Imager. A QIAquick PCR Purification Kit (Catalogue 28104, Qiagen, Germantown, MD, USA) was used to purify the amplified PCR products following the manufacturer’s protocol. Fifteen DNA fragments were selected and sent to Inqaba Biotechnical Industries Pty Ltd., Pretoria, Gauteng, South Africa, for sequencing. The fragments were chosen to represent different locations based on the quality of the purified DNA.

### 2.6. Sequencing and Phylogenetic Analysis

Sanger sequencing of the amplified products was carried out to confirm the identity of the detected virus. Obtained sequences were trimmed and assembled using Bioedit software version 7.7.1. Consensus sequences were then analyzed by BLAST+ version 2.17.0 (NCBI) to check for the identity of viruses. Multiple alignment was done with obtained sequences, together with other reference accessions of passion fruit potyviruses retrieved from the GenBank database using ClustalW in MEGA X (Table 1). GenBank accessions were selected based on the host crop (passion fruit) and the targeted sequence (coat protein). Retrieved sequences were edited to match the partial CP region obtained in the present study. Phylogenetic analyses of aligned sequences were performed using MEGA v.11 [18], with the evolutionary distances computed using the Unweighted Pair Group Method (UPGMA) with 1000 bootstrap replications [19]. Pairwise sequence comparisons were carried out on aligned sequences using Bioedit. The neighbor-joining tree was rooted in the sequence of squash vein yellowing virus (SqVYV) of the *Ipomovirus* genus (DQ812125.1). Sequences of the 15 new Rwandan isolates were logged in GenBank as accessions PX686122, PX686123, PX686124, PX686125, PX686126, PX686127, PX686128, PX686129, PX686130, PX686131, PX686132, PX686133, PX686134, PX686135 and PX686136.

## 3. Results

### 3.1. Incidence and Severity of the Viral Symptoms in Farmers’ Fields

**Figure 2:**
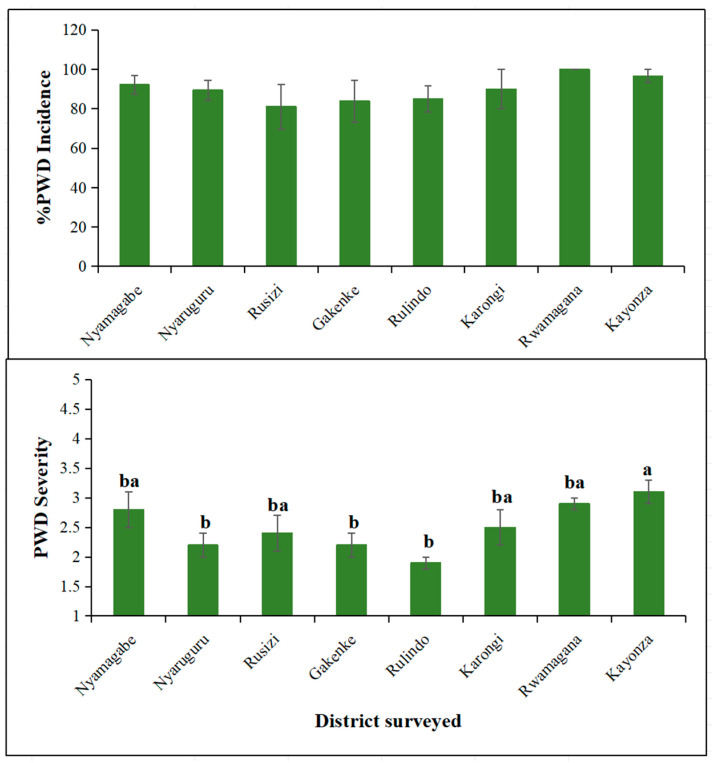
Percentage mean incidence (**top**) and severity (**bottom**) of viral disease symptoms per district. Bars represent means, and error bars represent standard error. Columns sharing the same letters are not significantly different according to ANOVA and Tukey tests (*p* < 0.05).

During the survey, common symptoms characteristic of passion fruit woodiness disease were observed, including mosaic patterns; blisters; distorted leaves; stunting; and small, hardened fruits that were sometimes cracked. The incidences of viral symptoms across the districts were not significantly different (*p* > 0.05) (Figure 2). Despite no significant difference, Rwamagana led, with 100%, followed by Kayonza (97%), Nyamagabe (92%), Karongi (90%), Nyaruguru (89%), Rulindo (85%), and Gakenke (84%), and the lowest incidence was in Rusizi district (81%). In contrast, the mean severity of viral symptoms differed significantly between the surveyed districts. Kayonza district led, with a severity score of 3.1, while Rulindo had the lowest severity score of 1.9. Rwamagana, Nyamagabe, Karongi and Rusizi had relatively similar severity scores, ranging from 2.9 to 2.4, in descending order. Viral disease prevalence was 100% in all surveyed areas.

### 3.2. Distribution of Viruses Determined by Serology and RT-PCR

Out of 216 leaf samples collected and tested using three ELISA kits (potyvirus, CABMV and CMV), 44% reacted positively to the generic potyvirus antibodies, and none reacted positively to CABMV or CMV (Table 2). Potyvirus infection was detected across all surveyed districts. Rusizi recorded the highest incidence of positive samples (82%), followed by Karongi (59%), Nyamagabe (56%), Rwamagana (52%), Nyaruguru (48%), Rulindo (26%), Gakenke (22%) and Kayonza (11%).

**Figure 3:**
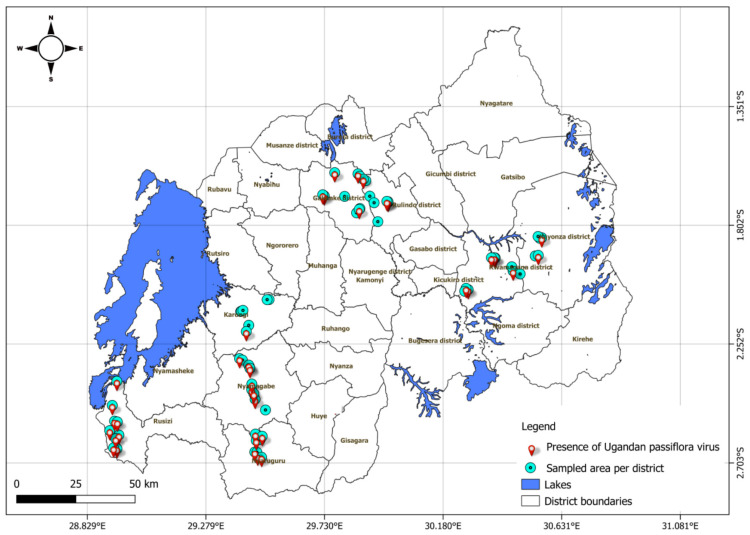
Map of Rwanda showing the presence and distribution of Ugandan passiflora virus based on results from reverse-transcription polymerase chain reaction in sampled districts.

Using RT-PCR, a total of 186 samples, including 96 determined positive and 90 determined negative by ELISA, were tested for the presence of UPV (Table 3). Of the 96 (52%) ELISA-positive samples, 74 (40%) tested positive for UPV infection, while 22 (12%) were negative. Among the 90 (48%) ELISA-negative samples, 29 samples (16%) tested positive for UPV. Total UPV infection was detected in 103 of 186 samples (55%), comprising 56% of the symptomatic (*n* = 126) and 53% of asymptomatic (*n* = 60) samples. Based on the number of samples tested per district, the prevalence of UPV infection in Rusizi was 89%, that in Rwamagana was 75%, that in Karongi was 74%, that in Nyamagabe was 59%, that in Nyaruguru was 44%, that in Kayonza was 38%, that in Gakenke was 30%, and that in Rulindo was 30%. The virus was present across all major passion fruit growing regions (Figure 3). Overall, out of the 216 collected samples, 125 (58%) samples tested positive using both ELISA and RT-PCR.

### 3.3. Sequences of Rwandan UPV Isolates

Fragments of the expected size (772 bp) were amplified from the tested samples using the UPVF2 and UPVR2 primers [14]. BLASTN search in the GenBank database showed that the 15 obtained sequences are closely related to previously isolated UPV isolates from passion fruit in Rwanda and Kenya. New coat-protein (CP) sequences were compared with one another and with UPV isolates previously reported in Rwanda, Uganda and Kenya and available in the NCBI database (Supplementary Material S1). New Rwandan UPV isolates (PX686122, PX686123, PX686124, PX686125, PX686126, PX686127, PX686128, PX686129, PX686130, PX686131, PX686132, PX686133, PX686134, PX686135, and PX686136) shared 97–99% nucleotide (nt) and 94–98% amino acid (aa) identities with one another and 89–94% nt with previously reported Rwandan UPV isolates (MK132862.1, MK132863.1, and MK132865.1). The comparison of new Rwandan isolates with other UPV isolates reported elsewhere revealed that Rwandan isolates shared 81–97% nt identities with Ugandan isolates (FJ896000.1, FJ896002.1, and FJ896003.1) and 80–82% with Kenyan isolates (MW355820.1, MW355822.1, MW355823.1, MW355830.1, and MW355831.1).

The genetic distance between the new (PX686122, PX686123, PX686124, PX686125, PX686126, PX686127, PX686128, PX686129, PX686130, PX686131, PX686132, PX686133, PX686134, PX686135, and PX686136) and previously isolated Rwandan isolates (MK132862.1, MK132863.1, MK132865.1) is very short, ranging between 0.01 and 0.03 (Supplementary Materials S2). Similarly, the distance from the new isolates to other Ugandan (FJ896002.1, FJ896003.1) and Kenyan (MW355820.1, MW355822.1, MW355823.1) UPV isolates was also low (0.01–0.03)—except for FJ896000.1 (Ugandan) and MW355830.1 and MW355831.1 (Kenyan), all of which had moderate distances of 0.21–0.22. Other reference isolates from Australia (JF427623.1 and JF427620.1), Japan (AB690439.1 and AB690447.1), Brazil (AY434454.1), USA (DQ860147.1) and the out-group (DQ812125.1) showed much larger genetic distances from all UPV isolates, ranging between 0.43 and 5.61.

### 3.4. Phylogenetic Relationships Between Rwandan, Ugandan, and Kenyan UPV Isolates and Other Representative Potyvirus Isolates

**Figure 4:**
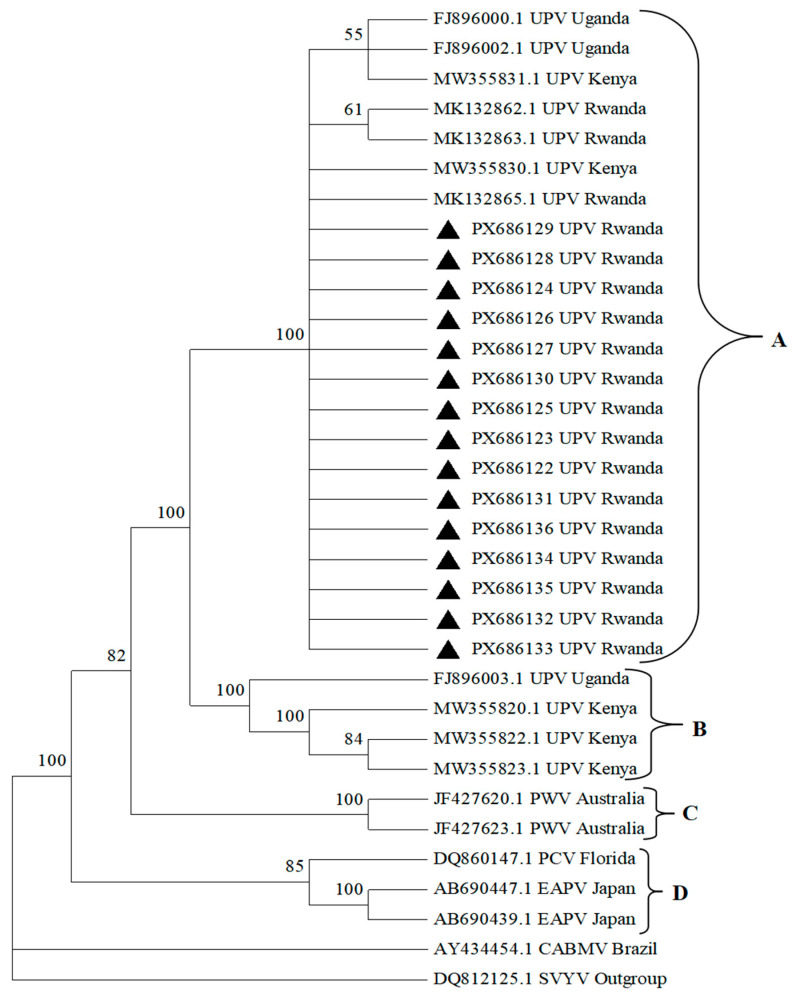
Phylogenetic tree constructed with sequences of 15 Ugandan Passiflora virus isolates, together with 17 other reference sequences of passion fruit viruses (Potyvirus genus) from different countries. The tree is based on alignments of 714 nucleotides of partial coat-protein regions and is rooted in the sequence of squash vein yellowing virus (SqVYV) (*Ipomovirus* genus; DQ812125.1). The accession numbers of the isolates and place of origin are indicated in the tree. Samples analyzed in the present study are indicated by the ▲ symbol. Clade A represent Ugandan passiflora isolates (Rwanda, Kenya, Uganda); Clade B represent Ugandan passiflora isolates (Rwanda and Uganda); Clade C represent passion fruit woodiness virus isolates (Australia); and Clade D represent East Asian Passiflora virus isolates (Japan).

Phylogenetic analysis of 15 UPV isolates based on partial sequences (714 bp) of the coat-protein region was done (Figure 4). The Rwandan isolates, including the new and previously reported isolates clustered together with some Uganda and Kenya isolates in a single major clade (A) that contained 15 new UPV isolates from Nyamagabe (PX686122, PX686123, and PX686124), Nyaruguru (PX686125 and PX686126), Gakenke (PX686127), Rulindo (PX686128, PX686129, and PX686130), Karongi (PX686131), Rwamagana (PX686132, PX686133, PX686134, and PX686135), and Kayonza (PX686136) districts, as well as previous Rwandan (MK132862.1, MK132863.1, and MK132865.1), Ugandan (FJ896000.1 and FJ896002.1) and Kenyan (MW355830.1 and MW355831.1) isolates. Clade B contained other UPV isolates reported in Kenya (MW355820.1, MW355822.1, and MW355823.1) and Uganda (FJ896003.1). Clade C contained PWV isolates from Australia (JF427623.1 and JF427620.1), and Clade D was composed of EAPV isolates from Japan (AB690439.1 and AB690447.1) and PCV from USA (DQ860147.1).

## 4. Discussion

Passion fruit is a priority cash crop for domestic and export markets in Rwanda [6]. However, its productivity is severely constrained by viral diseases in Rwanda, as well as in the East Africa region [10,12,14]. Findings from the present study show that passion fruit woodiness disease is widespread, with a prevalence of 100% in the surveyed locations. This finding is consistent with previous studies in Kenya that indicated a wide distribution of passion fruit woodiness disease among farmers’ fields [8,20]. Symptoms characteristic of passion fruit woodiness disease on fruits and leaf distortion were observed in all surveyed areas. A high incidence of viral disease (81 to 100%), coupled with significant differences in disease severity (1.9 to 3.1) across locations, is epidemiologically important. A similar pattern has been documented in passion fruit, with a very high disease incidence of up to 100% recorded in central regions of Uganda [10]. In addition, high incidences (70 to 100%) in passion fruit vines and disease severity in the range of 2.4 to 3.7 have been reported in Kenya [20]. High disease incidence and variations in severity could be due to differences in agro-ecological zones. Sampled areas in Kayonza and Rwamagana are located at low altitudes, while Gakenke, Rulindo, Karongi, Rusizi, Nyamagabe and Nyaruguru are in high-altitude zones. Thus, differences in environmental factors may have had an influence on vector population dynamics and viral transmission [20]. Low-altitude AEZs often support larger populations and increased activity of insect vectors such as aphids, which are major transmitters of plant viruses, including potyviruses [20].

Many tested samples were determined to be positive for potyviruses using the genus-specific monoclonal antibody, and further analysis with RT-PCR using UPV-specific primers confirmed the presence of UPV in the majority of these. These findings confirm that the virus was present in all surveyed districts, showing widespread occurrence. Similarly, a previous study reported UPV as the most prevalent virus in passion fruit fields in Rwanda causing woodiness disease [14]. The virus was initially reported in Uganda in 2018 and was most recently reported in Kenya in 2025, reinforcing its regional significance [10,12]. The wide distribution of the virus across the growing areas may be attributed to movement and use of diseased planting materials, as well as limited farmer knowledge, leading to poor management practices associated with the spread of the virus. Based on the results, it is evident that UPV is already established within the major local passion fruit production zones, indicating the need for the development of sustainable management strategies.

Nucleotide identity analysis showed high similarity (89–99%) among new and previous Rwandan isolates. It was also evident that there were strong similarities between the Rwandan isolates and previously reported isolates from Uganda and Kenya. Previous Rwandan isolates had 93–100% nt identities with Ugandan reference isolates, while Kenyan isolates had 81–99% nt identities with Ugandan isolates [12,14]. Such high sequence similarities suggests that the virus population is relatively homogenous. This is further supported by the close genetic relationship between Rwandan, Ugandan and Kenyan UPV isolates revealed by the short genetic distance observed in the present study. This suggests a common ancestry and a high possibility of movement of infected planting materials across borders, facilitating the spread of the same viral strain across the countries. This spread is further enhanced by local transmission mediated by aphid vectors. In addition, the clustering of the Rwandan, Ugandan and Kenyan UPV isolates into one major clade is an indication of regional spread or movement of viral strains. This concurs with a previous report indicating that cross-border movement of viruses via planting materials plays a key role in the introduction of viral isolates from one country to another [10].

About 11% of ELISA-positive potyvirus samples tested negative for UPV-specific RT-PCR. This is an indication of the possible presence of other potyviruses infecting passion fruit. Several potyviruses have been reported in association with passion fruit, including East Asian passiflora virus (EAPV), cowpea aphid-borne mosaic virus (CABMV), telosma mosaic virus (TeMV), and passion fruit woodiness virus (PWV) [15,21,22,23,24,25]. Considering the symptoms observed in the collected samples, more diagnostic assays targeting other viruses are needed. Conversely, RT-PCR showed higher sensitivity, detecting UPV in 16% of the samples that were identified as negative for potyvirus by ELISA. These results show that RT-PCR was more sensitive than ELISA and highlight the growing need for highly sensitive molecular diagnostics to accurately detect and differentiate potyvirus species in the context of increased global plant material exchange and stricter certification requirements.

On the other hand, almost half of the asymptomatic samples were infected with viruses. The detection of potyviruses and UPV in asymptomatic samples can be attributed to low virus titers, early stages of infection, and environmental conditions that suppress symptom expression, despite active viral replication [26,27]. Previous studies have documented such latent infections in passion fruit, as well as in other perennial crops, often revealed only through sensitive molecular techniques such as RT-PCR [28,29]. Asymptomatic but infected plants are epidemiologically significant because they can act as hidden reservoirs of inoculum, facilitating viral spread within and between fields [22]. This has important implications for farmers’ planting materials, as selecting seedlings based solely on the absence of visible symptoms can lead to the unintended spread of viral infections [8,30,31]. This emphasizes the need for certified virus-free materials and routine diagnostic screening to effectively manage viral diseases, as use of uncertified, infected planting materials poses a major risk to sustainable passion fruit production.

## 5. Conclusions

The widespread distribution of UPV across all surveyed passion fruit growing districts in Rwanda, with 100% prevalence and a high incidence rate (55%), demonstrates that the virus is well established in major growing areas. This poses a significant threat to sustainable passion fruit production in the country. The current findings also indicate that UPV isolates across East Africa are closely related, with a common ancestry, suggesting regional movement of infected planting material. The limited genetic variability observed among UPV isolates further implies that harmonized control and management approaches could be effectively implemented across the region.

To achieve sustainable management of UPV, the government should strengthen certification schemes for virus-free planting material and enhance farmer capacity through targeted training on virus recognition, vector control, and field sanitation practices. Strengthening extension services is also critical to ensure that farmers are adequately informed about viral disease management and the risks associated with propagating infected planting materials. Therefore, reinforcing seed and planting-material certification systems will be vital to limiting the spread of viral diseases that threaten income security among smallholder farmers.

Furthermore, given the evidence of potyvirus diversity and the likelihood of undetected pathogens, additional diagnostic assays targeting viruses beyond those analyzed in this study are strongly recommended. Further research should also focus on understanding the effects of mixed viral infections on symptom severity, yield losses, and overall crop performance. Finally, the development of integrated virus management strategies is necessary to safeguard passion fruit production and ensure long-term productivity in the region.

## Figures and Tables

**Table 1:** Known potyvirus isolate sequences retrieved from GenBank and used for sequence comparison.

No.	Virus	Isolate	Host	Origin	Accession No.
1	Ugandan passiflora virus	UGM-19a	Passion fruit	Uganda	FJ896000.1
2	Ugandan passiflora virus	UGM-73	Passion fruit	Uganda	FJ896002.1
3	Ugandan passiflora virus	UGM-17	Passion fruit	Uganda	FJ896003.1
4	Ugandan passiflora virus	RW10	Passion fruit	Rwanda	MK132862.1
5	Ugandan passiflora virus	RW23	Passion fruit	Rwanda	MK132863.1
6	Ugandan passiflora virus	RW140	Passion fruit	Rwanda	MK132865.1
7	Ugandan passiflora virus	UPV-KV	Passion fruit	Kenya	MW355820.1
8	Ugandan passiflora virus	UPV-KT	Passion fruit	Kenya	MW355822.1
9	Ugandan passiflora virus	UPV-KS	Passion fruit	Kenya	MW355823.1
10	Ugandan passiflora virus	UPV-KK	Passion fruit	Kenya	MW355830.1
11	Ugandan passiflora virus	UPV-KJ	Passion fruit	Kenya	MW355831.1
12	Passion fruit woodiness virus	PWV-BuW-1	Passion fruit	Australia	JF427623.1
13	Passion fruit woodiness virus	PWV-MuW-1	Passion fruit	Australia	JF427620.1
14	East Asian passiflora virus	EAPV-AT1	Passion fruit	Japan	AB690439.1
15	East Asian passiflora virus	EAPV-SY102	Passion fruit	Japan	AB690447.1
16	Cowpea aphid-borne mosaic virus	CABMV-M3	Passion fruit	Brazil	AY434454.1
17	Passiflora chlorosis virus	PCV	Passion fruit	USA	DQ860147.1
18	Squash vein yellowing virus	SqVYV	Squash		DQ812125.1

**Table 2:** Summary of samples tested for potyviruses using Enzyme-Linked Immunosorbent Assay (ELISA) across major passion fruit growing districts in Rwanda.

Test	District	Total Samples Collected	Symptomatic		Asymptomatic		Total Positive ELISA
			Number Tested	Potyvirus-Positive	Number Tested	Potyvirus-Positive	
ELISA	Nyamagabe	27	18	8	9	7	15 (56%)
	Nyaruguru	27	18	6	9	7	13 (48%)
	Rusizi	27	18	15	9	7	22 (82%)
	Karongi	27	18	12	9	4	16 (59%)
	Rulindo	27	18	5	9	2	7 (26%)
	Gakenke	27	18	4	9	2	6 (22%)
	Rwamagana	27	18	10	9	4	14 (52%)
	Kayonza	27	18	2	9	1	3 (11%)
	Total	216	144	62 (43%)	72	34 (47%)	96 (44%)

**Table 3:** Summary of Ugandan passiflora virus infection across different regions based on results from Reverse-Transcription Polymerase Chain Reaction (RT-PCR).

Test	District	Samples Collected	Samples Tested	ELISA ^+^ & UPV ^+^	ELISA ^+^ & UPV ^−^	ELISA ^−^ & UPV ^+^	Total UPV ^+^ Samples
RT-PCR	Nyamagabe	27	27	13	2	3	16 (59%)
	Nyaruguru	27	27	9	4	3	12 (44%)
	Rusizi	27	27	21	1	3	24 (89%)
	Karongi	27	27	16	0	4	20 (74%)
	Rulindo	27	27	2	5	6	8 (30%)
	Gakenke	27	27	0	6	8	8 (30%)
	Rwamagana	27	16	11	3	1	12 (75%)
	Kayonza	27	8	2	1	1	3 (38%)
	Total	216	186	74 (40%)	22 (12%)	29 (16%)	103 (55%)

ELISA—Enzyme-Linked Immunosorbent Assay; UPV—Ugandan passiflora virus; (+)—positive samples; (−)—negative samples.

## Data Availability

The data used to support the findings of this study are included within the article. Raw data will be made available upon request.

## Supplementary Materials

The following supporting information can be downloaded at: https://www.mdpi.com/article/10.3390/v18030397/s1, Supplementary Material S1: Nucleotide and deduced amino acids percentage identities of Rwandan isolates of the Ugandan passiflora virus in comparison to isolates from other countries; Supplementary Material S2: Genetic distance matrix showing evolutionary or sequence divergence of Rwandan, Ugandan and Kenyan isolates of the Ugandan passiflora virus and related potyvirus species.
