# Supplementary material for: Distribution of Ugandan Passiflora Virus (Potyvirus passiflorafricanse) in Major Passion Fruit Growing Areas in Rwanda
Source: Viruses. 2026 Mar 23;18(3):397. doi: 10.3390/v18030397 (PMC13030864; doi:10.3390/v18030397)
Supplement: Supplementary file 1 [file viruses-18-00397-s001.zip › viruses-4019872-supplementary.pdf]

MANUSCRIPT TITLE: Distribution of Ugandan passiflora virus (Potyvirus passiflorafricanse) in Major Passion fruit Growing Areas in Rwanda

Supplementary Material S1. Nucleotide and deduced amino acids percentage identities of Rwandan isolates of the Ugandan passiflora virus in comparison to isolates from other countries

No.	Isolate	1	2	3	4	5	6	7	8	9	10	11	12	13	14	15	16	17	18	19	20	21	22	23	24	25	26
**1**	**PX686123_Rwanda**	ID	0.98	0.96	0.97	0.95	0.96	0.96	0.96	0.95	0.98	0.95	0.96	0.96	0.94	0.96	0.94	0.95	0.59	0.87	0.92	0.92	0.56	0.57	0.57	0.79	0.78
**2**	**PX686122_Rwanda**	0.99	ID	0.95	0.97	0.96	0.97	0.97	0.96	0.95	0.99	0.95	0.96	0.97	0.95	0.97	0.94	0.95	0.58	0.87	0.91	0.91	0.56	0.57	0.57	0.79	0.78
**3**	**PX686124_Rwanda**	0.98	0.98	ID	0.96	0.96	0.95	0.95	0.96	0.95	0.95	0.95	0.95	0.95	0.94	0.94	0.93	0.93	0.58	0.87	0.91	0.90	0.56	0.57	0.56	0.78	0.78
**4**	**PX686125_Rwanda**	0.98	0.99	0.98	ID	0.96	0.97	0.97	0.97	0.96	0.97	0.96	0.96	0.96	0.95	0.96	0.94	0.94	0.59	0.87	0.92	0.91	0.56	0.57	0.57	0.79	0.79
**5**	**PX686126_Rwanda**	0.98	0.98	0.98	0.98	ID	0.96	0.96	0.96	0.95	0.96	0.95	0.96	0.96	0.95	0.95	0.94	0.94	0.59	0.88	0.92	0.91	0.57	0.57	0.57	0.78	0.78
**6**	**PX686127_Rwanda**	0.98	0.99	0.98	0.98	0.98	ID	0.97	0.97	0.96	0.97	0.96	0.97	0.96	0.95	0.97	0.94	0.95	0.59	0.86	0.91	0.91	0.57	0.58	0.58	0.79	0.79
**7**	**PX686128_Rwanda**	0.98	0.98	0.98	0.98	0.98	0.98	ID	0.97	0.96	0.97	0.96	0.97	0.96	0.96	0.97	0.95	0.95	0.59	0.88	0.92	0.92	0.57	0.58	0.57	0.79	0.79
**8**	**PX686130_Rwanda**	0.98	0.98	0.98	0.99	0.98	0.98	0.98	ID	0.96	0.96	0.98	0.98	0.97	0.96	0.96	0.94	0.94	0.58	0.87	0.91	0.92	0.56	0.57	0.56	0.79	0.79
**9**	**PX686129_Rwanda**	0.97	0.98	0.98	0.98	0.98	0.98	0.97	0.98	ID	0.94	0.95	0.95	0.95	0.94	0.95	0.93	0.94	0.57	0.86	0.91	0.91	0.56	0.57	0.57	0.79	0.79
**10**	**PX686131_Rwanda**	0.99	0.99	0.98	0.98	0.98	0.98	0.98	0.98	0.97	ID	0.95	0.96	0.97	0.95	0.97	0.95	0.95	0.59	0.87	0.92	0.92	0.57	0.58	0.57	0.79	0.78
**11**	**PX686132_Rwanda**	0.98	0.98	0.97	0.98	0.98	0.98	0.97	0.99	0.97	0.98	ID	0.98	0.97	0.97	0.95	0.93	0.93	0.58	0.86	0.90	0.91	0.56	0.57	0.56	0.78	0.78
**12**	**PX686133_Rwanda**	0.98	0.98	0.97	0.98	0.98	0.98	0.98	0.98	0.97	0.98	0.99	ID	0.98	0.98	0.97	0.94	0.95	0.59	0.86	0.91	0.92	0.57	0.57	0.57	0.79	0.79
**13**	**PX686135_Rwa**	0.9	0.9	0.9	0.9	0.9	0.9	0.9	0.9	0.9	0.9	0.9	0.9	ID	0.9	0.9	0.9	0.9	0.5	0.8	0.9	0.9	0.5	0.5	0.5	0.7	0.7

	**nda**	8	8	7	8	8	8	7	8	8	8	8	9		7	6	4	4	8	7	1	2	6	7	6	8	8
**14**	**PX686134_Rwa**	0.9	0.9	0.9	0.9	0.9	0.9	0.9	0.9	0.9	0.9	0.9	0.9	0.9	ID	0.9	0.9	0.9	0.5	0.8	0.9	0.9	0.5	0.5	0.5	0.7	0.7
	**nda**	7	8	7	8	8	8	7	8	7	8	8	9	8		5	3	4	8	6	0	1	6	7	7	7	7
**15**	**PX686136_Rwa**	0.9	0.9	0.9	0.9	0.9	0.9	0.9	0.9	0.9	0.9	0.9	0.9	0.9	0.9	ID	0.9	0.9	0.5	0.8	0.9	0.9	0.5	0.5	0.5	0.7	0.7
	**nda**	8	8	7	8	8	8	8	8	7	8	8	8	8	7		5	5	9	7	2	2	7	8	8	8	8
**16**	**FJ896000.1_Uga**	0.9	0.9	0.9	0.9	0.9	0.9	0.9	0.9	0.9	0.9	0.9	0.9	0.9	0.9	0.9	ID	1.0	0.5	0.8	0.9	0.9	0.5	0.5	0.5	0.8	0.8
	**nda**	7	7	7	7	8	8	7	7	7	7	7	7	7	7	7		0	9	9	3	3	7	8	8	0	1
**17**	**FJ896002.1_Uga**	0.9	0.9	0.9	0.9	0.9	0.9	0.9	0.9	0.9	0.9	0.9	0.9	0.9	0.9	0.9	1.0	ID	0.5	0.8	0.9	0.9	0.5	0.5	0.5	0.8	0.8
	**nda**	7	8	7	8	8	8	7	8	8	8	7	7	7	7	7	0		9	9	4	4	7	8	8	1	1
**18**	**FJ896003.1_Uga**	0.8	0.8	0.8	0.8	0.8	0.8	0.8	0.8	0.8	0.8	0.8	0.8	0.8	0.8	0.8	0.8	0.8	ID	0.5	0.5	0.5	0.6	0.7	0.7	0.4	0.4
	**nda**	1	1	1	1	2	2	1	2	1	1	1	1	1	1	1	2	2		2	6	7	9	0	0	4	3
**19**	**MK132862.1_R**	0.8	0.8	0.8	0.8	0.8	0.8	0.8	0.8	0.8	0.8	0.8	0.8	0.8	0.8	0.8	0.9	0.9	0.7	ID	0.9	0.9	0.5	0.5	0.5	0.7	0.7
	**wanda**	9	9	9	9	9	9	9	9	9	9	9	9	9	9	9	0	0	4		4	2	1	2	1	5	5
**20**	**MK132863.1_R**	0.9	0.9	0.9	0.9	0.9	0.9	0.9	0.9	0.9	0.9	0.9	0.9	0.9	0.9	0.9	0.9	0.9	0.7	0.9	ID	0.9	0.5	0.5	0.5	0.8	0.8
	**wanda**	3	4	4	4	4	4	3	4	4	4	3	3	3	3	3	5	5	8	4		8	4	5	5	0	0
**21**	**MK132865.1_R**	0.9	0.9	0.9	0.9	0.9	0.9	0.9	0.9	0.9	0.9	0.9	0.9	0.9	0.9	0.9	0.9	0.9	0.7	0.9	0.9	ID	0.5	0.5	0.5	0.8	0.8
	**wanda**	3	3	3	3	3	3	3	4	3	3	3	3	3	3	3	4	5	8	3	9		5	5	5	0	0
**22**	**MW355820.1_K**	0.8	0.8	0.8	0.8	0.8	0.8	0.8	0.8	0.8	0.8	0.8	0.8	0.8	0.8	0.8	0.8	0.8	0.8	0.7	0.7	0.7	ID	0.5	0.9	0.4	0.4
	**enya**	1	1	1	1	2	2	1	1	1	1	1	1	1	1	1	2	2	7	4	8	8		5	8	2	1
**23**	**MW355822.1_K**	0.8	0.8	0.8	0.8	0.8	0.8	0.8	0.8	0.8	0.8	0.8	0.8	0.8	0.8	0.8	0.8	0.8	0.8	0.7	0.7	0.7	0.9	ID	0.9	0.4	0.4
	**enya**	1	1	1	1	2	2	1	2	2	1	1	2	1	2	1	3	2	7	4	8	8	9		9	3	2
**24**	**MW355823.1_K**	0.8	0.8	0.8	0.8	0.8	0.8	0.8	0.8	0.8	0.8	0.8	0.8	0.8	0.8	0.8	0.8	0.8	0.8	0.7	0.7	0.7	0.9	1.0	ID	0.4	0.4
	**enya**	1	1	1	1	2	2	1	1	2	1	1	2	1	1	1	2	2	7	4	8	8	9	0		2	1
**25**	**MW355830.1_K**	0.8	0.8	0.8	0.8	0.8	0.8	0.8	0.8	0.8	0.8	0.8	0.8	0.8	0.8	0.8	0.8	0.8	0.6	0.7	0.8	0.8	0.6	0.6	0.6	ID	0.9
	**enya**	0	1	1	1	1	1	1	1	1	1	0	1	0	0	0	1	2	5	6	1	1	5	5	5		8
**26**	**MW355831.1_K**	0.8	0.8	0.8	0.8	0.8	0.8	0.8	0.8	0.8	0.8	0.8	0.8	0.8	0.8	0.8	0.8	0.8	0.6	0.7	0.8	0.8	0.6	0.6	0.6	0.9	ID
	**enya**	0	1	1	1	1	1	1	1	1	1	1	1	0	0	0	2	2	5	6	1	1	5	5	5	9	

Supplementary Material S2. Genetic distance matrix showing evolutionary or sequence divergence of Rwandan, Ugandan and Kenyan isolates of the Ugandan passiflora virus and related potyvirus species

Isolate	1	2	3	4	5	6	7	8	9	10	11	12	13	14	15	16	17	18	19	20	21	22	23	24	25	26	27	28	29	30	31
PX686123_UPV_Rwanda																															
PX686122_UPV_Rwanda	0.01																														
PX686124_UPV_Rwanda	0.02	0.02																													
PX686125_UPV_Rwanda	0.02	0.01	0.02																												
PX686126_UPV_Rwanda	0.02	0.02	0.02	0.02																											
PX686127_UPV_Rwanda	0.02	0.01	0.02	0.02	0.02																										
PX686128_UPV_Rwanda	0.02	0.02	0.02	0.02	0.02	0.02																									
PX686130_UPV_Rwanda	0.02	0.02	0.02	0.01	0.02	0.02	0.02																								
PX686129_UPV_Rwanda	0.03	0.02	0.02	0.02	0.02	0.02	0.03	0.02																							
PX686131_UPV_Rwanda	0.01	0.01	0.02	0.02	0.02	0.02	0.02	0.02	0.03																						
PX686132_UPV_Rwanda	0.02	0.02	0.03	0.02	0.02	0.02	0.03	0.01	0.03	0.02																					
PX686133_UPV_Rwanda	0.02	0.02	0.03	0.02	0.02	0.02	0.02	0.02	0.03	0.02	0.01																				

**PX686135_UPV_Rwanda**	0.02	0.02	0.03	0.02	0.02	0.02	0.03	0.02	0.02	0.02	0.02	0.01														
**PX686134_UPV_Rwanda**	0.03	0.02	0.03	0.02	0.02	0.02	0.03	0.02	0.03	0.02	0.02	0.01	0.02													
**PX686136_UPV_Rwanda**	0.02	0.02	0.03	0.02	0.02	0.02	0.02	0.02	0.03	0.02	0.02	0.02	0.02	0.03												
**FJ896000.1_UPV_Uganda**	0.03	0.03	0.03	0.03	0.02	0.02	0.03	0.03	0.03	0.03	0.03	0.03	0.03	0.03	0.03											
**FJ896002.1_UPV_Uganda**	0.03	0.02	0.03	0.02	0.02	0.02	0.03	0.02	0.02	0.02	0.03	0.03	0.03	0.03	0.03	0.00										
**FJ896003.1_UPV_Uganda**	0.21	0.22	0.21	0.21	0.21	0.21	0.22	0.21	0.22	0.22	0.22	0.21	0.22	0.22	0.22	0.20	0.20									
**MK132862.1_UPV_Rwanda**	0.01	0.01	0.01	0.01	0.01	0.02	0.01	0.01	0.01	0.01	0.02	0.02	0.02	0.02	0.02	0.00	0.00	0.21								
**MK132863.1_UPV_Rwanda**	0.02	0.02	0.02	0.01	0.01	0.02	0.02	0.02	0.02	0.02	0.02	0.02	0.02	0.02	0.02	0.01	0.01	0.20	0.00							
**MK132865.1_UPV_Rwanda**	0.02	0.02	0.02	0.02	0.02	0.02	0.02	0.02	0.02	0.02	0.02	0.02	0.02	0.02	0.03	0.01	0.01	0.20	0.01	0.01						
**MW355820.1_UPV_Kenya**	0.22	0.22	0.22	0.22	0.21	0.21	0.22	0.22	0.22	0.22	0.22	0.22	0.22	0.22	0.22	0.20	0.20	0.15	0.21	0.21	0.21					
**MW355822.1_UPV_Kenya**	0.22	0.22	0.22	0.22	0.21	0.21	0.22	0.21	0.21	0.22	0.22	0.21	0.22	0.21	0.22	0.20	0.20	0.14	0.21	0.20	0.21	0.01				
**MW355823.1_UPV_Kenya**	0.22	0.22	0.22	0.22	0.21	0.21	0.22	0.22	0.21	0.22	0.22	0.21	0.22	0.22	0.22	0.20	0.20	0.14	0.21	0.21	0.21	0.01	0.00			
**MW355830.1_UPV_Kenya**	0.02	0.02	0.02	0.01	0.02	0.01	0.02	0.02	0.02	0.02	0.02	0.02	0.02	0.02	0.02	0.01	0.01	0.24	0.01	0.01	0.01	0.24	0.24	0.24		
**MW355831.1_UPV_Kenya**	0.02	0.02	0.02	0.02	0.02	0.01	0.02	0.02	0.01	0.02	0.02	0.02	0.02	0.02	0.02	0.00	0.00	0.24	0.00	0.01	0.01	0.24	0.24	0.24	0.01	
**JF427620.1_PWV**	0.	0.	0.	0.	0.	0.	0.	0.	0.	0.	0.	0.	0.	0.	0.	0.	0.	0.	0.	0.	0.	0.	0.	0.	0.	0.

**_Australia**	43	44	44	44	43	44	43	44	43	44	44	44	44	44	44	44	43	46	43	44	44	43	43	44	43	44				
**AB690447.1_EAP**	0.	0.	0.	0.	0.	0.	0.	0.	0.	0.	0.	0.	0.	0.	0.	0.	0.	0.	0.	0.	0.	0.	0.	0.	0.	0.	0.			
**V_Japan**	47	48	47	47	47	47	47	47	47	48	48	48	48	48	47	48	47	50	47	47	48	48	49	48	48	47	51			
**DQ860147.1_PC**	0.	0.	0.	0.	0.	0.	0.	0.	0.	0.	0.	0.	0.	0.	0.	0.	0.	0.	0.	0.	0.	0.	0.	0.	0.	0.	0.	0.		
**V_Florida**	50	50	50	51	51	50	51	51	50	50	51	50	49	50	50	50	50	52	44	48	48	52	51	51	50	50	47	42		
**AY434454.1_CA**	1.	1.	1.	1.	1.	1.	1.	1.	1.	1.	1.	1.	1.	1.	1.	1.	1.	1.	1.	1.	1.	1.	1.	1.	1.	1.	1.	1.	1.	
**BMV_Brazil**	38	40	39	39	39	42	41	41	43	40	43	43	44	41	41	44	44	40	36	40	41	38	39	39	45	45	46	67	66	
**DQ812125.1_SV**	4.	5.	4.	5.	5.	5.	4.	5.	5.	5.	5.	5.	5.	5.	5.	5.	5.	5.	4.	4.	4.	5.	5.	5.	4.	4.	4.	4.	4.	4.
**YV**	38	16	35	12	17	12	41	26	22	12	08	20	21	13	23	12	10	07	07	02	05	19	19	11	34	37	26	23	29	15
